# Utility of Soluble CD163 in the Clinical Management of Patients With Kawasaki Disease

**DOI:** 10.3389/fped.2020.00148

**Published:** 2020-04-07

**Authors:** Yoshihiro Azuma, Yasuo Suzuki, Seigo Okada, Chie Matsuguma, Hiroyuki Wakiguchi, Yuji Ohnishi, Takashi Furuta, Akiko Miyake, Hiroki Yasudo, Kiyoshi Ichihara, Shouichi Ohga, Shunji Hasegawa

**Affiliations:** ^1^Department of Pediatrics, Yamaguchi University Graduate School of Medicine, Ube, Japan; ^2^Department of Laboratory Sciences, Faculty of Health Sciences, Yamaguchi University Graduate School of Medicine, Ube, Japan; ^3^Department of Pediatrics, Graduate School of Medical Sciences, Kyushu University, Fukuoka, Japan

**Keywords:** intravenous immunoglobulin, Kawasaki disease, macrophage, soluble CD163, sCD163

## Abstract

**Objective:** Intravenous immunoglobulin (IVIG) therapy is a useful first-line treatment for Kawasaki disease (KD); however, 10–20% of patients fail to respond and require additional IVIG. Soluble CD163 (sCD163) is considered a biomarker for macrophage activation. There are no reports measuring serum sCD163 in KD patients. This study aimed to explore its possible utility in the clinical management of patients with KD.

**Methods:** Eighty-seven patients with well-defined KD were retrospectively enrolled together with 19 healthy individuals with comparable ages. KD patients were classified into three groups, Group A (initial IVIG responders), Group B (additional IVIG responders), and Group C (additional IVIG non-responders). Serum sCD163 together with complete blood counts, C-reactive protein, d-dimer, albumin, aspartate aminotransferase (AST), and alanine aminotransferase (ALT) were measured before the initial IVIG treatment in all cases, and afterward in a fraction of cases.

**Results:** Serum sCD163 in KD patients before initial IVIG was generally much higher than the control group. The median (interquartile range) of sCD163 was as follows: Control 446 (385–521) ng/mL; Group A, 699 (478–1,072); Group B, 1,349 (1,116–1,390); and Group C, 665 (544–1,094). In general, sCD163 showed close positive correlation with ALT and AST, but none with other markers. Among the KD groups, Group B showed the highest sCD163: Group B vs. A: *p* = 0.0003; B vs. C: *p* = 0.035). Serum sCD163 was significantly increased after IVIG in Group A, while no change occurred in others.

**Conclusion:** The serum sCD163 levels could be a useful biomarker in the clinical management of KD, especially for predicting responsiveness to IVIG.

## Introduction

Kawasaki disease (KD) is an early childhood acute inflammatory illness characterized by prolonged fever, diffuse mucosal inflammation, indurative edema of the hands and feet, polymorphous skin rash, and non-suppurative lymphadenopathy (1). Although high-dose intravenous immunoglobulin (IVIG, 2 g/kg/dose) with oral aspirin (30 mg/kg/day) is the first-line therapy for patients with KD in Japan as per Japanese guideline for treatment of acute Kawasaki disease (2), 10–20% of patients with KD are resistant to initial IVIG treatment. Coronary artery lesions (CALs) are the most severe complications of KD, and IVIG non-responders have a higher risk of CALs than IVIG responders (3, 4). For predicting the initial IVIG response, several scoring systems including Gunma score, Osaka score, and Kurume score, have been published in Japan (5–7). Nevertheless, the feasibility of these scores varied among races (8). Repeat IVIG therapy for refractory cases has been applied in practice. However, there is little information on the use of a predictive marker for additional IVIG responsiveness. The active mechanisms of IVIG resistance in children with KD are not understood fully.

CD163 is a scavenger receptor for haptoglobin-hemoglobin, expressed on monocytes/macrophages (9). Shedding of CD163 is regulated by a disintegrin and metalloproteinase-17 that mediate shedding of tumor necrosis factor-α, a cytokine produced mainly by activated monocytes/macrophages (10). Reports show sCD163 as a useful biomarker for macrophage activation in several inflammatory diseases, such as sepsis, inflammatory bowel disease, and influenza-associated encephalopathy (11–13). We previously reported an increased number of peripheral CD14^+^CD16^+^ cells, activated monocytes/macrophages in acute KD, and activated monocytes/macrophages role in the pathophysiology of KD (14). Therefore, it is of interest to evaluate the pathophysiological implication of sCD163 in KD as a maker of excessive macrophage activation. So far, there has been no primary report that measured serum sCD163 in KD.

This study was conducted to explore clinical utility of sCD163 in the management of KD in comparison with other laboratory tests. From our recent clinical experiences in measuring sCD163 in KD, we had a specific interest in seeking its possible utility in predicting responsiveness to IVIG among KD patients.

## Materials and Methods

### Patients

The patients were diagnosed with KD following the 5th revision of Diagnostic Guidelines (15). KD was classified into complete or incomplete type. The complete type defined patients fulfilling 5/6 or all six major symptoms or 4/6 major symptoms combined with CAL as confirmed by coronary angiography or two-dimensional echocardiography, during the disease course. Incomplete type defined patients who did not fulfill the diagnostic criteria but suspected with KD, for example, a patient with only 4/6 major symptoms without CAL, and presence of other diseases excluded.

Acute-phase CALs defined as >3 mm expansion of coronary artery diameter in patients younger than 5 years old, or 1.5 times expansion compared with the coronary artery diameter at diagnosis in five or more years of age. If these changes did not normalize over one month of illness, it defined late-phase CALs. However, no patients suffered from the late-phase CALs in this study. We identified the patients as IVIG non-responders, who had a persistent or recrudescent fever (>37.5°C) at 48 h after IVIG infusion and who required additional therapies (16).

**Figure 1:**
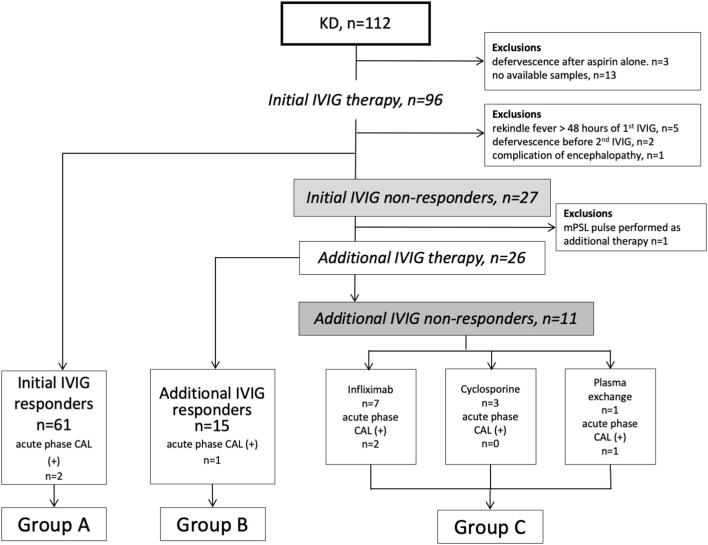
Flow chart of KD patients in this study. KD, Kawasaki disease; IVIG, intravenous immunoglobulin; CAL, coronary arterial lesion; mPSL, methylprednisolone.

This retrospective observational study included 112 patients with KD treated in Yamaguchi University Hospital between January 2005 and December 2015 (Figure 1). Twenty-five patients were excluded because of defervescence after aspirin therapy alone, unavailable acute-phase samples, febrile flare-up after more than 48 h of an initial IVIG, defervescence before additional-IVIG, a complication of encephalopathy, and received pulsed methylprednisolone as additional therapy. We analyzed 87 patients who received IVIG (2 g/kg/dose) with oral aspirin (30 mg/kg/day) as initial therapy. All initial IVIG non-responders received additional-IVIG (2 g/kg/dose) as the second-line therapy. Eleven patients required the third-line therapy (infliximab; 7, cyclosporine; 3, plasma exchange; 1) (Figure 1). Firstly, we dichotomized them into initial IVIG responders and initial IVIG non-responders. Secondarily, to analyze the patients in detail, we classified them into three subgroups. Group A composed of initial IVIG responders. Group B composed of patients who responded to the additional IVIG. Group C composed of patients who did not respond to the repeated IVIGs and required a third-line therapy (additional IVIG non-responders). Control subjects included 19 healthy children without febrile illness.

We measured serum sCD163 levels in patients during acute-phase of KD and collected other laboratory findings before initial IVIG and clinical data from the medical records at the time of KD diagnosis. Laboratory findings included complete blood count, serum levels of sodium, aspartate aminotransferase (AST), alanine aminotransferase (ALT), C-reactive protein (CRP), albumin, and plasma levels of d-dimer. Serum sCD163 were determined using an sCD163 enzyme-linked immunosorbent assay kit (R&D Systems, Minneapolis, MN, USA) with a detection limit of 1.56 ng/mL, following the manufacturer's instructions. Acute phase samples in all groups were collected within 2 days before the initial-IVIG start. For evaluation of temporal changes in laboratory parameters, serum sCD163 and related parameters were measured again, where possible, within 2 days after initial IVIG. As a limitation of retrospective nature of this study, the number of cases (n) that were valid for the paired analysis was 29 of 87 cases (Group A: *n* = 9 of 61, Group B: *n* = 10 of 15, Group C: *n* = 10 of 11). All serum samples for sCD163 were stored at −20°C after collection.

The Institutional Review Board at Yamaguchi University Hospital (H29-158) approved the retrospective study. Informed consent obtained from the parents of all the patients enrolled in this study.

### Statistical Analysis

Statistical analyses for comparison were by Fisher's exact probability test for the categorical data. For multiple comparisons among the three groups, the Kruskal-Wallis test and Steel-Dwass test were used for numerical data. Spearman's correlation coefficient (*r*_s_) was used to analyze associations between numerical parameters. Wilcoxon singed-rank test were performed for evaluating differences in paired data. *P*-value of <0.05 considered statistically significant. These statistical analyses were performed using JMP^®^ 13 (SAS Institute Inc., Cary, NC, USA).

## Results

Clinical characteristics of patients with KD are shown in Table 1. The number of patients with acute-phase CALs and high Gunma score (≥5 points) was significantly deferent in the three groups. There was no significant difference in febrile days until diagnosis in three groups.

**Table 1:** Clinical characteristics of KD patients.

	**Group A *n* = 61**	**Group B *n* = 15**	**Group C *n* = 11**	***p*-value**
Sex; male: female	33 : 28	12 : 3	9 : 2	0.067
Months of age at diagnosis, median, range	18 (1-121)	19 (7-52)	17 (1-61)	0.82
Febrile days until diagnosis, median, range	4 (2-9)	4 (3-6)	4 (2-8)	0.15
Disease type, complete: incomplete	55: 6	13: 2	11: 0	0.61
Acute phase CALs	2 (3%)	1 (6%)	3 (27%)	0.015^*^
Gunma score ≥5 points	15 (25%)	8 (53%)	8 (73%)	0.003^**^

*KD, Kawasaki disease; IVIG, intravenous immunoglobulin; CALs, coronary arterial lesions; Group A, initial-IVIG responders; Group B, additional-IVIG responders; Group C, patients who require 3rd-line therapy*,

* *p < 0.05*,

** *p < 0.01*.

**Figure 2:**
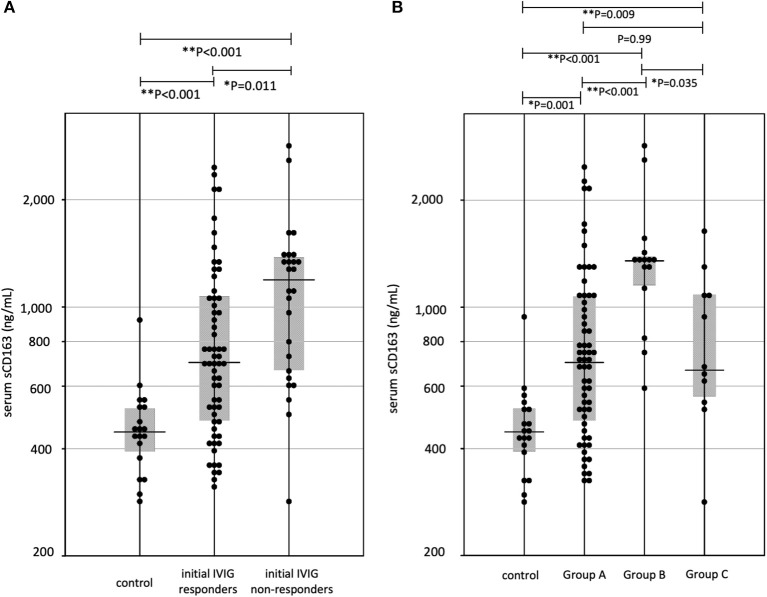
**(A)** The sCD163 levels in Kawasaki disease (KD). IVIG, intravenous immunoglobulin, ^*^P < 0.05, ^**^P < 0.01 **(B)** The relationship three groups divided for effective therapies and sCD163 levels. Group A, initial IVIG responders; Group B, additional IVIG responders; Group C, patients who require 3rd line therapy, ^*^P < 0.05, ^**^P < 0.01.

Median (interquartile range) of serum sCD163 levels in initial-IVIG non-responders was 1,269 (665–1,385) ng/mL, higher than initial-IVIG responders: 699 (478–1,072) ng/mL (Figure 2A).

Figure 2B shows serum levels of sCD163 in the three groups. Median (interquartile range) values of serum sCD163 levels were, Group A: 699 (478–1,072) ng/mL, Group B: 1,349 (1,116–1,390) ng/mL, and Group C: 665 (544–1,094) ng/mL. The median level in Group B was the highest among all three groups, however the levels did not differ between Groups A and C. Clinical variables in acute-phase were compared among the three groups. The *p*-values were as follows, sCD163: *P* < 0.001, WBC: *P* = 0.87, hemoglobin: *P* = 0.70, platelets: *P* = 0.03, AST: *P* = 0.002, ALT: *P* = 0.019, CRP: *P* = 0.21, sodium: *P* = 0.001, albumin: *P* = 0.15. For further analysis, we compared laboratory data among each group by Steel-Dwass method (Table 2). The serum AST levels of Group B was highest in three groups; however, they did not differ between Groups A and C. Serum levels of sCD163, AST, and ALT showed significant positive correlations (sCD163 vs. AST: *r*_s_ = 0.41, *P* < 0.001, sCD163 vs. ALT: *r*_s_ = 0.48, *P* < 0.001, and AST vs. ALT: *r*_s_ = 0.88, *P* < 0.001). Other laboratory parameters were not significantly correlated with sCD163.

**Table 2:** Comparison among the three groups.

	**Group A**	**Group B**	**Group C**	***P*****-value**		
	***n* = 61**	***n* = 15**	***n* = 11**	**A vs. B**	**A vs. C**	**B vs. C**
sCD163	699 (478–1,072)	1,349 (1,116–1,390)	665 (544–1,094)	0.001^**^	0.99	0.035^*^
WBC (×10^9^/L)	12.76 (10.18–16.59)	13.01 (9.98–15.22)	11.96 (7.84–16.84)	0.96	0.90	0.87
Monocyte (×10^9^/L)	8.18 (5.14–11.44)	8.91 (4.49–9.35)	4.41 (1.42–10.27)	0.97	0.19	0.56
Hemoglobin (g/dL)	11.4 (10.9–12.0)	11.6 (11.0–12.5)	11.1 (10.8–11.8)	0.85	0.87	0.67
Platelets (×10^9^/L)	355 (304–406)	342 (289–371)	263 (239–331)	0.27	0.061	0.31
AST (IU/L)	38 (27-78)	95 (55–423)	27 (24-39)	0.002^**^	0.70	0.030^*^
ALT (IU/L)	24 (12-71)	106 (48–405)	20 (10-168)	0.001^**^	0.11	0.24
CRP (mg/dL)	5.9 (3.6–8.7)	7.1 (4.8–10.6)	7.8 (3.9–12.7)	0.42	0.32	0.99
Sodium (mEq/L)	135 (133–136)	133 (132–135)	132 (130–134)	0.094	0.004^**^	0.22
Albumin (g/dL)	3.8 (3.5–4.1)	3.7 (3.3–3.7)	3.6 (3.1–4.0)	0.49	0.16	0.65
D-dimer (μg*/*mL)	1.6 (1.3–2.4)	2.2 (1.5–2.2)	2.1 (1.1–3.3)	0.47	0.40	0.92

*WBC, white blood cell; AST, aspartate transaminase; ALT, alanine transaminase; CRP, C-reactive protein. Group A, initial-IVIG responders; Group B, additional-IVIG responders; Group C, patients who required 3rd-line therapy*,

* *p < 0.05*,

** *p < 0.01*.

**Figure 3:**
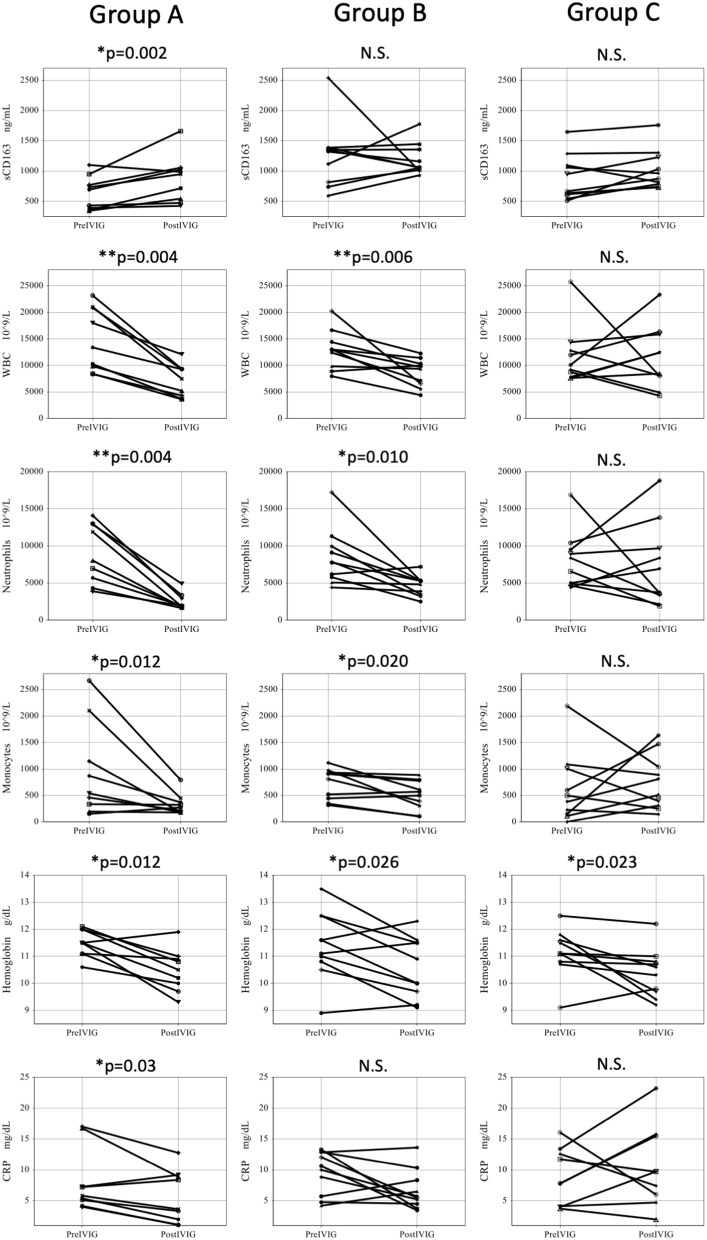
Comparison of parameters before and after initial IVIG among three groups. Pre IVIG, before initial Intravenous immunoglobulin; Post IVIG, after initial intravenous immunoglobulin; Group A, initial IVIG responders; Group B, additional IVIG responders; Group C, patients who require 3rd line therapy; WBC, white blood cell; N.S., not significant, ^*^P < 0.05, ^**^P < 0.01.

Figure 3 shows comparison of parameters before and after initial IVIG among IVIG responders and IVIG non-responders. In Group A, the serum sCD163 significantly increased after IVIG, while WBC, neutrophil counts, monocyte counts, hemoglobin, and CRP significantly decreased. In Group B, sCD163 did not show changes after the initial IVIG, while WBC, neutrophil counts, and hemoglobin decreased significantly. In contrast, in Group C, neither sCD163 nor other parameters showed any change except for slight lowering of hemoglobin.

## Discussion

In the present study, we first revealed that the serum levels of sCD163, a macrophage activation marker, are elevated in patients with acute-phase KD, and indicated that serum sCD163 can be used as a predictive marker for IVIG responsiveness.

The increased number and activation of CD14^+^CD16^+^ monocytes/macrophages via nuclear factor-κB (NF-κB) pathway during acute KD supported our previously published results (14, 17, 18).

We evaluated serum levels of monocyte chemotactic protein-1 (MCP-1) as an activation marker for monocytes/macrophages using the same samples; however, there were no significant differences among groups A: 517 (59–1,311) pg/mL, B: 765 (338–1,129) pg/mL, and C: 633.1 (302–1,609) pg/mL. (Supplementary Figure 1). These results suggest that sCD163 is obviously more useful in predicting additional IVIG responsiveness, compared with other monocytes/macrophages activation markers, such as MCP-1.

In present study, serum sCD163 showed correlation with AST and ALT. Liver function abnormalities have been reported as possible markers for predicting IVIG resistance (19). The mechanism of liver abnormality in KD is not clear. However, it has been reported that Kupffer cells, acting as liver macrophage, proliferate and/or swell in liver of acute KD patients (20). These findings suggest that the liver function abnormality is a result of hyperactivation of macrophages.

After the initial IVIG, significant reduction was observed for WBC and CRP among the initial IVIG responders. These results match with those previously reported (21). Regarding post-treatment changes in sCD163, initial IVIG responders (Group A) showed significant elevation. In contrast, initial IVIG non-responders (Group B and C) did not show any change, but monocyte counts were decreased significantly. This discrepancy may be explained by presumable detachment of CD163 on the macrophage surface induced by IVIG treatment in Group A. In support for this assumption, Samuelsson et al. reported that IVIG treatment causes morphological change in Fcγ receptor on macrophages (22). Furthermore, Sulahian et al. (23) reported that cross-linking of Fcγ receptors triggered shedding of CD163 from macrophage surface via ADAM17. On the other hand, changes in sCD163 were observed neither in Group B nor in Group C, which may reflect the failure of IVIG to act on macrophages for induction of any therapeutic effect.

In the comparison between Group B and C regarding the paired changes, WBC, neutrophil, and monocyte decreased significantly in Group B, but not in Group C. This discrepancy is attributable to the fact that Group B had higher levels of serum sCD163 before IVIG compared to Group C. These findings imply that the profiles of immunological responses are diverse among the IVIG resistance cases. Although the detailed therapeutic mechanism of IVIG is unclear, several *in vitro* studies showed IVIG inhibition of monocyte/macrophage activation controlled by the NF-κB pathway (24, 25). A retrospective study in China showed elevated serum tumor necrosis factor-α levels in KD, especially in IVIG non- responders (26). These studies suggested an over-activation of macrophages as one of the mechanisms for IVIG-resistant KD. In contrast, several studies showed T cell activation in the pathophysiology of IVIG-resistant KD. CD8^+^ T cells were present in the media of coronary artery aneurysm by pathological findings (27). Recently, we reported the association of human leukocyte antigen (HLA)-DR expressing T cells with IVIG-resistance in children with KD (28). Excessive CD8^+^ T cell activation and an imbalance in CD8^+^ T cell activation and inhibition contribute to the pathogenesis of KD (29). In this setting, in addition to the hyperactivation of macrophages, other mechanisms may account for the pathophysiology of IVIG-resistance in KD.

Previous predictive scores, such as Gunma score, Osaka score, and Kurume score, were constructed for predicting initial IVIG responsiveness. Although several treatments are proposed for initial IVIG non-responders, additional IVIG is most common in the United States and Japan (4, 30). However, at present additional IVIG or infliximab is the treatment choice for initial IVIG non-responders. For this reason, the prediction of additional IVIG responsiveness may provide utility as a second-line treatment. A prospective study in China suggests serum procalcitonin as a marker for predicting additional IVIG responsiveness (31), however there are few reports regarding potential marker for predicting additional IVIG responsiveness.

On the other hand, for predicting initial IVIG responsiveness, there have been reports on the utility of white blood cell count, hemoglobin, CRP, albumin, and d-dimer (32–34). However, we failed to predict the responsiveness of additional IVIG by those conventional markers as shown in Table 2. The present study suggests that serum sCD163 before initial IVIG may be useful as a predicting marker for additional IVIG responsiveness among patients non-responsive to initial IVIG, although further study is needed to confirm this hypothesis. The present study has some limitations. Firstly, the study population was small, and all recruited patients were Japanese. Regional or ethnic differences are associated with the pathophysiology of KD (8). Therefore, additional surveys are required to confirm these results in other ethnic groups. Secondly, we did not consider which therapy is suitable for each additional IVIG non-responder case, because the present study was a retrospective study. The choice of third-line treatment for additional IVIG non-responders was entrusted to the discretion of each pediatrician.

## Conclusion

In conclusion, this study clearly showed that serum levels of sCD163, macrophage activation marker, are elevated in patients with acute-phase KD. After initial IVIG, sCD163 was increased in initial IVIG-responders but not in non-responders. Close correlation of serum sCD163 with ALT and AST suggest that liver function abnormality commonly seen in KD may be a result of macrophage activation. Among the initial IVIG non-responders, sCD163 was higher in patients who responded to the additional IVIG. This finding indicates diversity of immunological profiles among the IVIG resistant cases and may be used for predicting the failure to the additional IVIG.

## Data Availability Statement

The raw data supporting the conclusions of this article will be made available by the authors, without undue reservation, to any qualified researcher.

## Ethics Statement

This study was carried out in accordance with the Declaration of Helsinki and the recommendations of the institutional review board of Yamaguchi University Hospital. The Institutional Review Board at Yamaguchi University Hospital (H29-158) approved the retrospective study. Informed consent obtained from the parents of all the patients enrolled in this study.

## Author Contributions

YA, YS, and SH were the principal investigators taking primary responsibility for the manuscript. YA, YS, SOh, and SH designed this study. SOk, CM, HW, YO, TF, and AM performed the clinical management with helpful discussion for the completion of the study. YA and YS took responsibility for the diagnosis and data collection. KI completed the statistical analysis. YA, YS, HY, and SH wrote the paper.

### Conflict of Interest

The authors declare that the research was conducted in the absence of any commercial or financial relationships that could be construed as a potential conflict of interest.

## Supplementary Material

Supplementary Figure 1 **(a)** The monocyte chemotactic protein-1 levels in Kawasaki disease (KD). IVIG: intravenous immunoglobulin **(b)** The relationship three groups divided for effective therapies and monocyte chemotactic protein-1 levels. Group A, initial IVIG responders; Group B, additional IVIG responders; Group C, patients who require 3rd line therapy.
