# Supplementary figures and images for: Utility of Soluble CD163 in the Clinical Management of Patients With Kawasaki Disease
Source: Front Pediatr. 2020 Apr 7;8:148. doi: 10.3389/fped.2020.00148 (PMC7154120; doi:10.3389/fped.2020.00148)

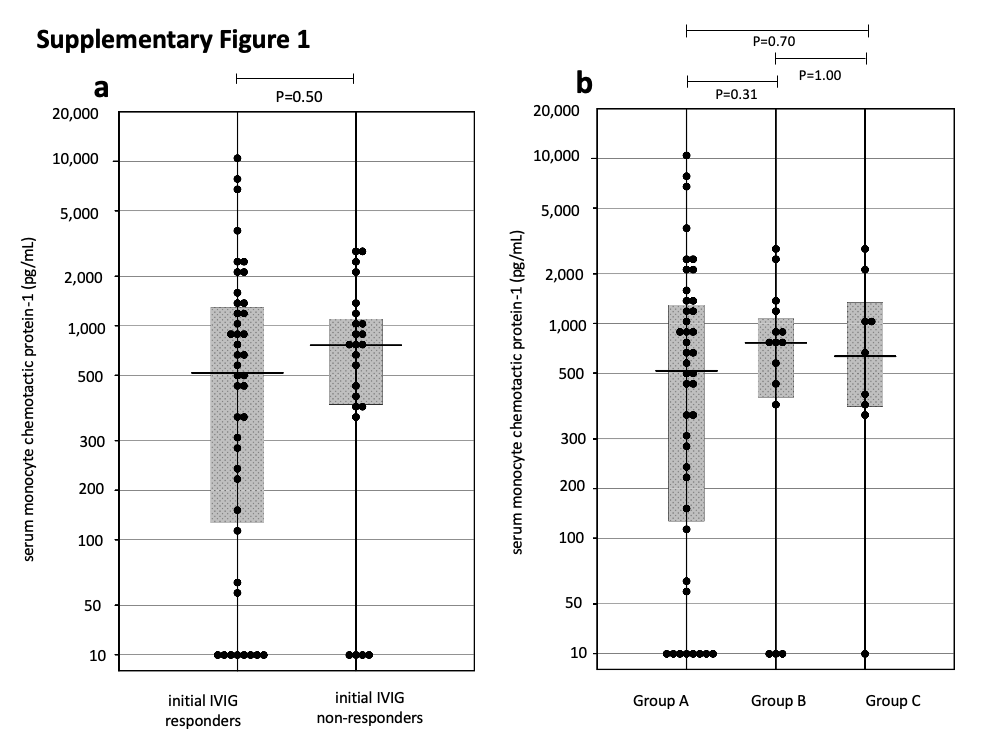

Supplement: Supplementary Figure 1 — (a) The monocyte chemotactic protein-1 levels in Kawasaki disease (KD). IVIG: intravenous immunoglobulin (b) The relationship three groups divided for effective therapies and monocyte chemotactic protein-1 levels. Group A, initial IVIG responders; Group B, additional IVIG responders; Group C, patients who require 3rd line therapy. [file Image_1.tiff]
